# Broadening the scope: Increasing phenotype diversity in laterality research

**DOI:** 10.3389/fnbeh.2022.1048388

**Published:** 2022-10-28

**Authors:** Lena Sophie Pfeifer, Katrin Heyers, Gesa Berretz, Dorothea Metzen, Julian Packheiser, Sebastian Ocklenburg

**Affiliations:** ^1^Department of Cognitive Psychology, Faculty for Psychology, Institute of Cognitive Neuroscience, Ruhr University Bochum, Bochum, Germany; ^2^Department of Biopsychology, Faculty for Psychology, Institute of Cognitive Neuroscience, Ruhr University Bochum, Bochum, Germany; ^3^Experimental Psychology II and Biological Psychology, Institute of Psychology, School of Human Sciences, Osnabrück University, Osnabrück, Germany; ^4^Social Brain Lab, Netherlands Institute for Neuroscience, Amsterdam, Netherlands; ^5^Department of Psychology, MSH Medical School Hamburg, Hamburg, Germany; ^6^Institute for Cognitive and Affective Neuroscience, MSH Medical School Hamburg, Hamburg, Germany

**Keywords:** laterality, lateralization, hemispheric asymmetries, social neuroscience, social touch, embracing, hugging

## Introduction

Research on functional brain lateralization made tremendous advances in the last decade (Ocklenburg et al., 2021), but shallow phenotyping is a continuing problem in large-scale studies using existing data. To specify, we understand shallow phenotyping as a minimal approach to assess phenotypes where the accuracy of the measurement is diminished and does not satisfy the complexity of the matter. For example, instead of using a full questionnaire or behavioral tool, the phenotype is assessed by a single item. This is often the case for e.g., handedness, which is one of the most prominent lateralized phenotypes in humans, with 89.4% of the population being right-handers (Papadatou-Pastou et al., 2020). Handedness is a complex trait emerging from multiple genetic, epigenetic, environmental, and interacting effects (Güntürkün and Ocklenburg, 2017; Ocklenburg et al., 2017; Kovel and Francks, 2019; Cuellar-Partida et al., 2021; Odintsova et al., 2022). About 25% of variance in human handedness has a genetic origin (Medland et al., 2006). Large-scale genome-wide-association studies (GWAS) have shown that handedness is a highly polygenic trait (Kovel and Francks, 2019; Wiberg et al., 2019; Cuellar-Partida et al., 2021). This indicates that many genes with small effect sizes contribute to handedness. In the largest GWAS, single nucleotide polymorphisms (SNPs) explained between 3.45% and 5.9% of variance in handedness (Cuellar-Partida et al., 2021). However, GWAS require enormous sample sizes, which is not feasible for most institutions that want to investigate genetic underpinnings of laterality. Fortunately, the summary statistics of GWAS can be used to calculate polygenic scores (PGS) in smaller sample sizes (Dudbridge, 2013). PGS are sum scores calculated from allelic effects of thousands of SNPs and suit as an indicator of genetic predisposition for a certain phenotype (Choi et al., 2020). It has been shown that PGS can predict handedness in a smaller independent sample (Ocklenburg et al., 2022).

Since a large part of the variance in handedness cannot be explained by genetic variation, epigenetic factors likely come into play (Schmitz et al., 2017). A recent epigenome-wide association study (EWAS) investigated the association between handedness and several hundred thousand cytosine-phosphate-guanine nucleotide base pairings (CpGs) from whole-blood samples (Odintsova et al., 2022). Methylations of two regions were significantly associated with left-handedness: BLCAP and IAH1. The study also reported that CpGs located near SNPs associated with handedness were more strongly associated with left-handedness than other CpGs. However, effect sizes were small and explained little of the variance in handedness.

One environmental factor that has been proposed to influence hemispheric asymmetries is stress (Ocklenburg et al., 2016). Stress has multiple effects on the organism and can result in mental and physical disorders (McEwen, 1998; Pfeifer et al., 2021). In reaction to stress exposure, the body initiates a stress response driven by two major systems: the sympathetic–adrenal–medullary (SAM) complex (Mason, 1968) and the hypothalamic–pituitary–adrenal (HPA) axis (Aguilera, 2011). The end product of the HPA axis - the hormone cortisol - has been associated with cognitive and behavioral adaptations under stress (Vogel et al., 2016) but also with various disorders (de Kloet et al., 2005; Zorn et al., 2017; Zänkert et al., 2019). It has been proposed that maternal stress affects offspring lateralization by means of epigenetic processes in humans and rodents (Schmitz et al., 2017). Similarly, birth stress has been associated with non-right-handedness (Bakan, 1971; Hicks et al., 1980). Unraveling relations between stress and lateralization is highly relevant since several mental disorders feature atypical asymmetries (Berretz et al., 2020b) while stress is a crucial factor for the development and progression of psychopathology (Cohen et al., 2007). Studies focusing on the effect of acute stress on cognitive laterality are still rare. In a study by Brüne et al. (2013), participants displayed increased asymmetric response tendencies in a dot probe task using face stimuli after stress induction. A recent study with rats demonstrated an increase in asymmetric turning behavior under high stress (Mundorf et al., 2020). However, only rats who experienced early life stress *via* separation from their mothers as pups showed this effect. In a recent series of studies by our group, we could not find an effect of acute stress or stress hormones on indicators of language and emotional lateralization on the behavioral level (Berretz et al., 2020a, 2021). While acute stress led to more left-hemispheric activity during stress induction itself (Berretz et al., 2022b), it did not affect basic interhemispheric transfer afterwards (Berretz et al., 2022c). These inconsistencies in results indicate that the relationship between acute stress and changes in functional hemispheric asymmetries may be more complex (Berretz and Packheiser, 2022). In this context, focusing on other forms of lateralized behavior like social touch could constitute a worthwhile avenue to pursue (Malatesta et al., 2020; Berretz et al., 2022a).

## Opinion: Broaden the scope - Research on the genetic and epigenetic key players in laterality needs more phenotypes than just handedness and language lateralization

Taken together, both genetic and epigenetic factors have a significant but small influence on human handedness, and there is a large gap of unexplained variance in the literature. We suggest that it is crucial to not only look into the ontogenetic factors, but also consider the phenotype when trying to understand this issue (Ocklenburg et al., 2014). Almost all large-scale studies on behavioral lateralization focus on handedness. Human handedness, however, is not an optimal phenotype to investigate either evolutionary, genetic or epigenetic questions in the broader scope of comparative laterality research for several reasons. The same is likely true for language lateralization, another widely used laterality phenotype (Hausmann et al., 2019). Importantly, both handedness and language lateralization are largely human specific. While limb preferences exist in many mammalian and non-mammalian species, animals often show individual-level asymmetry, but no population-level asymmetry (Ströckens et al., 2013; Ocklenburg et al., 2019; Manns et al., 2021). Even in those species that do show a significant population-level asymmetry for limb preferences, the left-right distribution is not as strongly skewed as in humans (Papadatou-Pastou et al., 2020). Still, discrepancies such as existing or missing population-level asymmetry that become apparent from a comparative perspective might also stimulate the question why laterality patterns differ across species and which factors play a role in exerting such differential influences. Another natural drawback of handedness or limb preferences in comparative laterality research is that it can only be observed in species that have limbs and use them to manipulate the environment.

We suggest that incorporating social laterality phenotypes (Marzoli et al., 2022) in studies on the ontogenesis and evolution of hemispheric asymmetries would benefit research in several ways. Laterality in social interactions has been found across many behavioral dimensions (Ocklenburg et al., 2018). These include walking side-by-side (Rodway and Schepman, 2022), hugging (Packheiser et al., 2019a), kissing (Ocklenburg and Güntürkün, 2009; Chapelain et al., 2015), and cradling children (Malatesta et al., 2019, 2020, 2021a; Packheiser et al., 2019b). Interestingly, studies show that these behaviors are all correlated with handedness, but only to a small to moderate extent in contrast to strong correlations with other forms of motor laterality, like footedness (Packheiser et al., 2020).

Importantly, social laterality can be observed in a wider range of species than limb preferences and is more comparable across species. For example, a wide variety of mammals show a side bias in mother-infant interactions (Karenina et al., 2017; Giljov et al., 2018; Karenina and Giljov, 2018). Moreover, fish schools show increased behavioral lateralization when predatory pressure is high, but fish are typically difficult to investigate regarding limb preferences, as they rarely use their fins to manipulate objects (Chivers et al., 2016). Several insect species such as bees or ants have also shown the need for social coordination in lateralized behavior (Anfora et al., 2011; Frasnelli et al., 2012, 2014; Rogers et al., 2013; Niven and Frasnelli, 2018) similar to the coordination needed during hugs and kisses in humans (Chapelain et al., 2015; Ocklenburg et al., 2018). This suggests that social laterality is phylogenetically old and conserved (Niven and Bell, 2018). Thus, social laterality could be a more suitable target behavior to uncover the mechanistic and genetic underpinnings of brain lateralization.

In addition to this benefit, social laterality may be a better phenotype for research on the evolution of laterality for another reason. In principle, two forms of evolutionary pressures to develop an asymmetrically organized nervous system exist. Firstly, there is an evolutionary pressure to develop an asymmetrically organized system *per se*, as it is more efficient and saves energy and neuronal tissue (Güntürkün and Ocklenburg, 2017). This form of evolutionary is independent of the direction, e.g., a leftward asymmetric function would be as beneficial for survival as a rightward asymmetric function. Secondly, there are also “social” evolutionary pressures for all individuals in a group (e.g., a shool of fish) to develop a lateral bias to the same side (Vallortigara, 2006). The direction of these biases becomes highly relevant when, for example, fleeing predators, as a single animal going to the other side than the rest of the group is easy prey (Vallortigara and Rogers, 2020). Thus, unlike the first form of evolutionary pressure to develop asymmetry, this second form is highly selective for direction of asymmetry. Importantly, handedness underlies the first form of evolutionary pressure, but it is debatable to what extent it underlies the second. For social laterality it is, however, rather clear that group coordination is highly relevant and empirical evidence shows that social pressure to go to one side does influence social laterality within groups (Chapelain et al., 2015). Thus, integrating social laterality phenotypes into research on the ontogenesis and evolution of hemispheric asymmetries could be highly beneficial as it likely underlies stronger evolutionary pressure than handedness, making it a behavioral phenotype that may have a stronger biological link to brain structure, which is determined by genetic and non-genetic factors.

## Integrating social laterality: A research proposal

While a few studies have studied the influence of stress on social laterality (Suter et al., 2007; Reissland et al., 2009) to this day, not a single study has investigated social laterality in the context of genetics and epigenetics. Thus, we believe that it is critical to include them in future large-scale investigations to uncover the genetic and epigenetic mechanisms of structural and functional brain lateralization. As extremely high sample sizes are needed to study the relationship between genes and behavior, we therefore propose to fill the gap in the literature by integrating especially behavioral measures of social laterality into test batteries for population-based genetic profiling such as the UK Biobank. Behavioral social laterality phenotypes can be easily acquired by asking participants to imagine, for example, to cradle a child, which has been demonstrated to reliably produce the universally found left-sided cradling bias (Malatesta et al., 2021b; Vauclair, 2022) or imagine to which side they turn their heads during a kiss. Such methods are highly economical as they can be included in survey questionnaires. We furthermore propose that comparative animal studies should be conducted to have mechanistic insight into the role of stress and determine the genetic basis of social laterality. This complementary approach to study social laterality will thus inform about potential genetic loci through human genome-wide association studies that can then be causally investigated in animal models through modern transgenic approaches such as CRISPR/Cas (Pickar-Oliver and Gersbach, 2019). Importantly however, we argue that the inclusion of social phenotypes would also advance laterality research when not combined with genetic or epigenetic approaches. Since one can assume that asymmetrically organized social behavior results from a principal functional lateralization of the brain, it may give insights into the hemispheric division of functions such as emotion processing and social cognition (Karenina and Giljov, 2018). With that, social laterality phenotypes may further shed light onto the groundwork of lateralization and its representation in the brain linking a range of behavioral asymmetries. As an alternative to genetic and epigenetic markers, studies on social laterality may consider the integration of imaging techniques such as electroencephalography (EEG) (e.g., Packheiser et al., 2021).

## Author contributions

LSP and KH wrote the section on stress. GB wrote the section on stress and laterality. DM wrote the section on genetic analyses. JP wrote the section on social laterality. SO conceptualized the article, wrote the first outline of the article, and the opinion section. All authors have read and critically revised the final submitted version of the article.

## Funding

The contribution of LSP was supported by the Deutsche Forschungsgemeinschaft (DFG, German Research Foundation) within project B4 of the Collaborative Research Center (SFB) 874 Integration and Representation of Sensory Processes (project-ID 122679504 SFB 874). KH was funded by the Deutsche Forschungsgemeinschaft (DFG, German Research Foundation) - project number GRK-2185/1 (DFG Research Training Group Situated Cognition)/Gefördert durch die Deutsche Forschungsgemeinschaft (DFG) - Projektnummer GRK-2185/1 (DFG-Graduiertenkolleg Situated Cognition). JP was supported by the German National Academy of Sciences Leopoldina (LPDS 2021-05).

## Conflict of interest

The authors declare that the research was conducted in the absence of any commercial or financial relationships that could be construed as a potential conflict of interest.

## Publisher's note

All claims expressed in this article are solely those of the authors and do not necessarily represent those of their affiliated organizations, or those of the publisher, the editors and the reviewers. Any product that may be evaluated in this article, or claim that may be made by its manufacturer, is not guaranteed or endorsed by the publisher.
